# Mental disorders in the Postpartum Period in Rio de Janeiro 2021–2023: *Nascer no Brasil* II Study

**DOI:** 10.11606/s1518-8787.2025059006527

**Published:** 2025-10-20

**Authors:** Mariza Miranda Theme-Filha, Marcia Leonardi Baldisserotto, Ana Claudia Santos Amaral, Maria Pappaterra Bastos, Arthur Orlando Correa Schilithz, Silvana Granado Nogueira da Gama, Karina de Cássia Caetano, Maria do Carmo Leal

**Affiliations:** I Fundação Oswaldo Cruz. Escola Nacional de Saúde Pública Sérgio Arouca. Departamento de Epidemiologia e Métodos Quantitativos em Saúde. Rio de Janeiro, RJ, Brasil; II Universidade Federal do Rio de Janeiro. Instituto de Psicologia. Departamento de Psicometria. Rio de Janeiro, RJ, Brasil; III Fundação Oswaldo Cruz. Instituto Nacional de Infectologia Evandro Chagas. Rio de Janeiro, RJ, Brasil

**Keywords:** Mental Disorders, Postpartum Period, Depression, Anxiety, Stress Disorders, Post-Traumatic

## Abstract

**OBJECTIVE:**

To analyze the prevalence and interrelationship of symptoms of depression, anxiety, and birth-related post-traumatic stress disorder.

**METHODS:**

Data from a cohort of postpartum women from the Nascer no Brasil II study, representative of births that occurred in the state of Rio de Janeiro between 2021 and 2023, were analyzed. Participants were interviewed face-to-face in the immediate postpartum period and again by telephone two months after birth. Women who responded to all questions on the Edinburgh Postnatal Depression Scale, Generalized Anxiety Disorder 7, and City Birth Trauma Scale were included, whereas pregnancies that ended in miscarriage or stillbirth were excluded, resulting in a total of 1,752 postpartum women. To test the homogeneity of proportions, the chi-square test (χ²) was used, with p-values below 5% considered statistically significant. The analysis of the interrelationships among the three symptoms was conducted using structural equation modeling (SEM), employing the weighted least squares mean and variance adjusted (WLSMV) estimator and theta parameterization.

**RESULTS:**

The prevalence of symptoms of depression, anxiety, and birth-related post-traumatic stress disorder was 17.9, 16.3, and 7.7%, respectively. A positive score on at least one of the scales was found in 24.6% of participants, and the simultaneous occurrence of two or three comorbidities was 12% and 3.7%, respectively. Low educational attainment and a history of mental disorders were significantly more prevalent in all three conditions analyzed. Structural equation modeling (SEM) revealed a significant and positive association among the three scales, and all latent variables in the model showed items with factor loadings greater than 0.5

**CONCLUSION:**

The postpartum period is critical for the diagnosis of mental disorders and may involve complex conditions in which symptoms of depression, anxiety, and stress overlap. It is important that healthcare professionals be aware of the occurrence and co-occurrence of these disorders, as well as their potential consequences for the health of both the woman and the newborn.

## INTRODUCTION

The birth of a baby can be one of the most rewarding experiences in a woman's life, but it is also a period of great vulnerability due to the major physical and emotional changes that occur during pregnancy and postpartum. Even without complications, this period is potentially stressful, increasing the risk of developing symptoms of mental disorders^1,2^. Globally, maternal mental disorders are considered a major public health challenge. In 2012, the World Health Organization (WHO) identified perinatal mental health, during pregnancy and up to one year postpartum, as a global priority, and improving the quality of maternal health is part of the Millennium Development Goals and the Sustainable Development Goals^4^. Mental health problems during this period mainly manifest as symptoms of depression, anxiety, and birth-related post-traumatic stress. The prevalence of these conditions varies depending on the stage of the pregnancy-postpartum cycle when they are assessed, the methods of assessment (self-reported or clinical interview), and the socioeconomic and cultural context of the studied population^5^.

Postpartum depression symptoms are the most common, with an average prevalence of 15.5% in high-income countries^6^ and reaching rates above 25% in middle- and low-income countries^7^. In Brazil, national data reveal that 26.3% of women exhibit symptoms of depression up to 12 months after childbirth^8^.

The prevalence of anxiety symptoms is similar to that of depression. About 20% of women report anxiety symptoms during the perinatal period, with higher frequency during pregnancy compared to the postpartum period^9^. A systematic review including studies from various socioeconomic contexts revealed an average prevalence of self-reported symptoms of 29.2% during the prenatal period, and 24.4% during the postnatal period. However, clinically diagnosed cases range from 8.1% in the prenatal period to 16% in the postpartum period^7,10^.

Symptoms of post-traumatic stress disorder (PTSD) associated with childbirth are related to the woman's negative experiences and perceptions of labor, which she interprets as a threat to her own life or the life of the baby^11^. PTSD affects between 3% and 6% of mothers, and among those who experienced obstetric complications, it may reach 19%^12^. As with symptoms of depression and anxiety, prevalence varies according to socioeconomic profile, with average rates exceeding 8% in low- and middle-income countries^7^.

Postpartum women with symptoms of depression, anxiety, and birth-related post-traumatic stress disorder share several risk factors, as well as overlapping symptoms that can influence each other in various ways, presenting interrelationships among them^13–15^. The main risk factors encompass various domains, such as socioeconomic, biological, psychosocial, and obstetric factors, with particular emphasis on previous and perinatal mental disorders, complications during labor and delivery, negative childbirth experiences, and lack of social support^16–18^.

Since women in the postpartum period may present with complex and overlapping symptoms, it is important to understand the prevalence and simultaneous occurrence of these conditions, recognizing that maternal mental health has a significant impact on parent-infant bonding, child development, and family functioning. Our hypothesis is that postpartum women who screen positive for depressive symptoms also screen positive for anxiety and post-traumatic stress symptoms.

In Brazil, population-based studies on the prevalence of maternal mental disorders in the postpartum period are scarce. Specifically, there are no studies on the interrelationship between symptoms of depression, anxiety, and postpartum post-traumatic stress disorder associated with childbirth. The aim of this study was to fill this gap by analyzing a cohort of Brazilian postpartum women interviewed immediately after childbirth and two months after delivery, based on data from the *Nascer no Brasil* II study (NBII - Birth in Brazil II study), a national survey on abortion, childbirth, and birth.

## METHODS

The NBII uses a probabilistic sample representative of hospital births in Brazil, with approximately 20,000 postpartum women admitted for childbirth (live or stillbirth) or abortion care. Data were collected from 2021 to 2024.

The sample was conducted with two stages of selection, corresponding to maternity hospitals and postpartum women, and stratified according to the country's macroregions (North, Northeast, Southeast, South, Central-West); type of hospital (public/mixed/private); location (capital cities and municipalities located in metropolitan regions and other municipalities); and the number of live births per year (100–499 live births/year, ≥ 500 live births/year). In addition to the face-to-face interview with postpartum women at the maternity ward, on average six hours after childbirth/abortion, the study included data collection from medical records, prenatal cards, and ultrasound examination results. It covers various socioeconomic and behavioral aspects, prenatal care, admission to the maternity ward, labor, childbirth, and immediate postpartum, as well as the circumstances of abortion and procedures performed. Two follow-up interviews were conducted via telephone at two and four months after childbirth/abortion with the women who participated in the baseline interview at the maternity ward. These interviews addressed the presence of maternal and neonatal morbidity, use of postnatal health services, breastfeeding, satisfaction with childbirth and abortion care, symptoms of depression, anxiety, and postpartum PTSD related to childbirth, obstetric violence, mother-infant bonding, and everyday discrimination. More details about the NBII research methodology are available in Leal et al.^19^ and Theme Filha et al.^20^.

For the state of Rio de Janeiro, a representative sample of births occurring between 2021 and 2023 was drawn, totaling 1,923 postpartum women. In the present analysis, data from the baseline and the first follow-up interview by phone were used, excluding abortions (n = 161) and stillbirths (n = 10), totaling 1,752 postpartum women. Since it was not possible to follow up with all women from the baseline (response rate of 64%), a logistic regression model was adjusted to estimate the probability of each woman responding to the phone interview, based on a set of variables that differentiated respondents from non-respondents, through propensity score. This procedure aimed to compensate for the tendency of women with certain characteristics not responding to the phone interview, assuming that they would have provided, on average, similar responses to the respondents within each stratum and adjustment category.

Variables related to the place of delivery, sociodemographic factors, and obstetric characteristics were tested. The most parsimonious logistic model included statistically significant variables (p < 0.05) that differentiated respondents from non-respondents. Non-respondents had less than 16 years of education, delivered in public hospitals (compared to private and mixed hospitals), and in hospitals with less than 500 births per year. The main reasons for non-response were incorrect phone numbers and failure to answer calls. Refusals were lower than 5%. Since it was a complex sample, in addition to weights, the design effect was corrected.

In the analysis of the prevalence of depression, anxiety, and postpartum PTSD symptoms, women who answered all the questions on the scales were included (response rate above 96% for the three scales), along with their respective 95% confidence intervals, considering the valid cutoff points for Brazil. To test the homogeneity of proportions, the χ2 test was performed, with p-values below 5% considered significant.

To assess the symptoms of depression, anxiety, and postpartum PTSD, the Edinburgh Postnatal Depression Scale (EPDS), Generalized Anxiety Disorder 7 (GAD7), and City Birth Trauma Scale (City BiTS) were used, respectively, two months after childbirth.

The EPDS^21^ contains 10 items that assess perinatal depressive symptoms, with a total score ranging from 0 to 30. The scale was validated for use in telephone interviews in Brazil, and a cutoff score of ≥ 10 indicates possible postpartum depression^22^.

The GAD7^23^ scale consists of seven items that assess anxiety symptoms, with four response options based on a Likert scale ranging from 0 (not at all) to 3 (almost every day), with a maximum score of 21 points. It was translated into Brazilian Portuguese^24^, and a cutoff score of ≥ 10 suggests the presence of moderate/severe anxiety symptoms.

City BiTS^25^, in its full version, contains 29 items that assess postpartum PTSD according to the definitions in the fifth edition of the Diagnostic and Statistical Manual of Mental Disorders (DSM-5)^26^ addressing the dimensions of intrusion, avoidance, and mood alteration. In this study, the 20 items that assess traumatic stress symptoms were used in the descriptive and bivariate analyses. The scale is valid for use in Brazil^27^, and a cutoff score of > 28 indicates a positive score for the Brazilian context^28^. In the structural equation modeling analyses, the factorial model proposed by the authors^25^ was used, consisting of two factors: Factor 1, which includes nine items related to postpartum PTSD symptoms, and Factor 2, which includes 10 items related to general stress symptoms.

The following variables were analyzed: sociodemographic characteristics (age, education level, race/skin color, marital status); obstetric history (parity, previous abortion or stillbirth); history of mental disorders (report of at least one of the following conditions: anxiety, depression, PTSD, schizophrenia, obsessive-compulsive disorder, bipolar disorder); timing of pregnancy (whether the woman felt the pregnancy happened at the right time, not quite the right time, or at the wrong time); type of pregnancy (singleton or twin); severe maternal morbidity; type of delivery (vaginal/forceps or C-section with or without labor); presence of a companion during hospitalization; payment method for childbirth (Brazilian Unified Health System — SUS / private health insurance / out-of-pocket); and newborn conditions (birth weight and gestational age, admission to neonatal intensive care unit). The variable severe maternal morbidity considered the presence of at least one criterion of potentially life-threatening condition or severe maternal morbidity, as defined by the WHO^29^.

The analysis of the interrelationships between symptoms of depression, anxiety, and postpartum PTSD was conducted using structural equation modeling (SEM). The weighted least squares mean and variance adjusted (WLSMV) estimator and theta parameterization were used. From these analyses, standardized estimated regression coefficients, their respective standard errors, and associated p-values were obtained to assess the associations between the model variables. These parameters were estimated using a bootstrap strategy with five thousand samples. A p-value below 0.05 was adopted to indicate statistical significance.

Model fit was assessed using the comparative fit index (CFI), Tucker-Lewis index (TLI), and root mean square error of approximation (RMSEA). Values of CFI and TLI greater than 0.95 and RMSEA lower than 0.06 were considered indicative of a good model fit. For RMSEA, a 90% confidence interval (90%CI) was used, with an acceptable lower bound close to 0 and an upper bound up to 0.08^30,31^. All statistical analyses were performed using the Statistical Package for the Social Sciences R version 3.5.1 and MPlus version 8.

### Ethical Aspects

The NBII study was approved by the National Research Ethics Commission (CONEP), under opinion number 3.909.299, on March 11, 2020.

## RESULTS

Table 1 highlights that the majority of respondents were between 20 and 34 years old (71.3%; 95%CI 68.3–74.1), reported brown skin color/race (50.2%; 95%CI 45.6–54.9), had between 9 and 15 years of education (68.7%; 95%CI 63.5–73.4), and were living with a partner (83.1%; 95%CI 80.4–85.6). Regarding obstetric variables, 46.5% (95%CI 42.7–50.3) had one or two previous births, and a history of abortion or stillbirth was reported by 20.6% (95%CI 16.8–24.9). A high proportion reported previous mental disorder (12.8%; 95%CI 10.8–15.1) and an unplanned pregnancy (35.4%). Pregnancy was reported as not quite at the right time by 26.6% (95%CI 21.4–32.5), and 8.8% (95%CI 6.8–11.2) considered it to have occurred at the wrong time. Medical records registered only 12 cases of twin pregnancies, and 14.2% (95%CI 11.1–18.1) of the postpartum women experienced severe complications during labor or delivery. Continuous presence of a companion throughout hospitalization was reported by 70.7% (95%CI 60.3–79.3), whereas only 5.9% (95%CI 2.5–13.4) had no companion at any point. Elective C-section without labor was the mode of delivery for 46.6% (95%CI 33.9–59.8) of the women, and 70% of hospitalizations were covered by SUS. The vast majority of newborns were in good health, with low birth weight and prematurity rates around 10%, and 6.2% (95%CI 4.0–9.6) were admitted to intermediate care or neonatal intensive care units (NICU).

**Table 1:** Sample description. *Nascer no Brasil* II study. Rio de Janeiro, 2021–2023.

Variables		Sample (n = 1,752)	
		n	(95%CI)
Age group (years)			
< 20		171	9.8 (7.3–12.9)
20–34		1.249	71.3 (68.3–74.1)
≥ 35		333	19.0 (15.9–22.5)
Skin color/race			
	White	512	29.2 (24.9–33.9)
	Black	360	20.6 (16.9–24.8)
	Brown	880	50.2 (45.6–54.9)
Education level			
	≤ 8	254	14.5 (11.2–18.7)
	9–15	1.204	68.7 (63.5–73.4)
	≥ 16	294	16.8 (12.7–21.9)
Marital status			
	Living with a partner	1.454	83.1 (80.4–85.6)
	Not living with a partner	295	16.9 (14.4–19.6)
Parity			
	Primiparous	747	42.6 (37.7–47.7)
	One or two previous labors	815	46.5 (42.7–50.3)
	Three or more previous labors	190	10.8 (9.3–12.6)
Previous abortion/stillbirth			
	Yes	360	20.6 (16.8–24.9)
	No	1.392	79.4 (75.1–83.2)
Previous mental disorder			
	Yes	224	12.8 (10.8–15.1)
	No	1.528	87.2 (84.9–89.2)
Timing of pregnancy occurred			
	At the right time	1.036	64.6 (60.0–69.0)
	Not exactly at the right time	426	26.6 (21.4–32.5)
	At the wrong time	141	8.8 (6.8–11.2)
Type of pregnancy			
	Singleton	1.740	99.3 (98.5–99.7)
	Twin	12	0.7 (0.3–1.5)
Severe maternal morbidity			
	Yes	250	14.2 (11.1–18.1)
	No	1.502	85.8 (81.9–88.9)
Presence of a companion during labor/delivery			
	In all moments	1.239	70.7 (60.3–79.3)
	At some moments	409	23.4 (17.9–29.9)
	No	104	5.9 (2.5–13.4)
Type of delivery			
	Vaginal or forceps	703	40.1 (33.6–47.1)
	C-section without labor	816	46.6 (33.9–59.8)
	C-section with spontaneous or induced labor	232	13.3 (7.5–22.4)
Payment for hospitalization			
	Unified Health System/public	1.288	73.6 (61.1–83.2)
	Health insurance plan	370	21.2 (12.3–33.8)
	Out-of-pocket payment	92	5.3 (2.3–11.8)
Weight at birth			
	< 1,500 g	16	0.9 (0.3–2,5)
	1,500–2,499	142	8.1 (6.3–10.5)
	≥ 2,500 g	1.591	90.9 (88.4–93.0)
Gestational age at birth (weeks)			
	< 37	183	10.4 (7.9–13.8)
	37 to 41	1.549	88.4 (84.6–91.4)
	≥ 42	20	1.1 (0.3–4.0)
Newborn admitted to a neonatal intensive care unit			
	Yes	109	6.2 (4.0–9.6)
	No	1.643	93.8 (90.4–96.0)

95%CI: 95% Confidence Interval.

Table 2 shows that the prevalence of symptoms of depression, anxiety, and birth-related PTSD was 17.9% (95%CI 14.6–21.9), 16.3% (95%CI 13.7–19.2), and 7.7% (95%CI 5.5–10.5), respectively. A positive score on at least one of the scales was found in 24.6% (95%CI 21.6–27.9) of the women, while the simultaneous occurrence of two or three comorbidities was 12% (95%CI 10.3–13.9) and 3.7% (95%CI 3.0–4.6), respectively. The prevalence of co-occurring symptoms of depression and anxiety was 10.5% (95%CI 8.8–12.5), and of depression and PTSD, 4.2% (95%CI 3.6–5.1). The co-occurrence of anxiety and PTSD symptoms reached 4.7% (95%CI 3.9–5.6).

**Table 2:** Prevalence of symptoms of depression, anxiety, post-traumatic stress associated to childbirth and comorbidities. *Nascer no Brasil* II Study. Rio de Janeiro, 2021–2023^a^.

Variables	n	% (95%CI)
Symptoms of depression	304	17.9 (14.6–21.9)
Symptoms of anxiety	275	16.3 (13.7–19.2)
Symptoms of post-traumatic stress associated to childbirth	129	7.7 (5.5–10.5)
At least one positive scale	432	24.6 (21.6–27.9)
Two positive scales	210	12.0 (10.3–13.9)
Three positive scales	65	3.7 (3.0–4.6)
Symptoms of depression and anxiety	184	10.5 (8.8–12.5)
Symptoms of depression and post-traumatic stress associated to childbirth	74	4.2 3.6–5.1)
Symptoms of anxiety and and post-traumatic stress associated to childbirth	82	4.7 (3.9–5.6)

a Symptoms of depression: score ≥ 10 at the Edinburgh Postnatal Depression Scale (EPDS); symptoms of anxiety: score ≥ 10 in the Generalized Anxiety Disorder 7 (GAD 7); symptoms of post-traumatic stress associated to childbirth: score > 28 in the City Birth Trauma Scale (City BiTS).

95%CI: 95% confidence interval.

Table 3 shows that postpartum women with low educational attainment (less than eight years of schooling) and a history of previous mental disorders had statistically higher prevalences of all three disorders analyzed, compared to the other categories of these variables.

**Table 3:** Prevalence of symptoms of depression, anxiety and post-traumatic stress associated to childbirth according to sociodemographic variables, clinical and obstetric history, labor/delivery and birth conditions. *Nascer no Brasil* II study. Rio de Janeiro, 2021–2023.

Variable		Symptoms of depression^b^ (n = 304)		P value	Symptoms of anxiety^c^ (n = 275)		P value	Sintomas de PTSD^d^ (n = 129)		P value
		n^a^	% (95%CI)		n^a^	% (95 %CI)		n^a^	% (95 %CI)	
Mother's age (years)										
	< 20	35	21.4 (17.2-26.3)	0.019	36	22.2 (16.3-29.5)	0.003	13	7.8 (4.9-12.4)	0.913
	20-34	235	19.5 (15.5-24.1)		211	17.5 (14.3-21.4)		90	7.5 (5.7-9.8)	
	≥ 35	33	10.3 (5.6-18.2)		27	8.3 (4.9-13.9)		26	8.1 (3.9-16.0)	
Skin color/race										
	White	70	14.2 (10.7-18.6)	0.170	50	10.2 (5.8-1 7.3)	0.014	33	6.7(4.0-11.0)	0.463
	Black	75	21.6 (15.7-28.9)		67	19.1 (15.9-22.8)		24	9.7 (6.0-15.2)	
	Brown	159	18.6 (13.5-25.1)		158	18.6 (15.8-21.7)		63	7.4 (4.9-11.1)	
Years of education										
	≤ 8	60	25.0 (19.1-32.0)	0.001	64	26.9 (20.8-34.1)	< 0.001	32	13.3 (7.7-21.9)	0.004
	9-15	219	18.6 (14.1-24.3)		191	16.2 (13.2-19.8)		92	7.9 (5.8-10.6)	
	≥ 16	24	8.7 (5.7-13.0)		20	7.2 (4.4-11.5)		6	2.0 (0.6-6.0)	
Marital status										
	Living with a partner	245	17.4(14.3-21.1)	0.307	213	15.2 (12.7-18.0)	0.114	113	8.1 (5.2-12.4)	0.332
	Not living with a partner	57	19.8 (14.1-27.1)		60	20.9 (14.1-30.0)		14	4.9 (2.4-9.9)	
Parity										
	Primiparous	126	1 7.6 (13.5-22.5)	0.315	105	14.6 (11.2-19.0)	0.169	36	5.0 (3.5-7.2)	0.030
	One or two previous labors	133	16.7(11.4-23.8)		113	14.3 (7.3-26.0)		66	8.4 (6.1-11.6)	
	Three or more previous labors	45	24.7 (16.7-34.9)		57	31.2 (1 8.4-47.7)		27	14.9 (6.3-31.1)	
Previous abortion/stiIIbirth										
	Yes	72	20.4 (14.5-27.9)	0.188	64	18.2 (13.5-24.1)	0.276	41	11.7(6.8-19.4)	0.012
	No	232	1 7.3 (14.3-20.7)		211	15.7 (13.4-18.5)		88	6.6 (4.8-9.0)	
Previous mental disorder										
	Yes	78	36.5 (26.2—48.2)	0.004	66	30.9 (1 9.0—45.9)	0.028	35	16.4 (12.2-21.7)	< 0.001
	No	225	15.2 (10.9-20.8)		209	14.1 (10.5-18.7)		94	6.4 (4.2-9.6)	
Timing of pregnancy occurred										
	At the right time	166	16.6 (13.2-20.6)	0.135	150	14.9 (12.4-1 7.8)	0.058	80	7.9 (5.8-10.8)	0.515
	Not exactly at the right time	88	21.5 (16.8-27.2)		87	21.7 (18.0-25.8)		40	10.1 (3.9-23.5)	
	At the wrong time	31	22.6 (12.7-36.9)		25	18.6 (10.3-31.4)		7	4.9 (1.8-12.5)	
Type of pregnancy										
	Singleton	298	17.7(14.3-21.7)	0.053	2 70	16.1 (13.6-19.1)	0.214	124	7.4 (5.2-10.4)	< 0.001
	Twin	6	45.8 (17.2-77.3)		4	33.2 (9.7-69.7)		6	45.8 (1 7.2-77.3)	
Severe maternal morbidity										
	Yes	50	20.4 (14.3-28.2)	0.306	54	22.1 (15.8-29.9)	0.024	38	16.1 (7.5-31.2)	0.008
	No	253	17.5 (14.2-21.4)		220	15.3 (12.9-18.0)		91	6.3 (5.1-7.8)	
Presence of a companion during labor/ delivery										
	In all moments	207	1 7.4 (14.8-20.3)	0.665	191	16.0 (13.7-18.7)	0.909	84	7.0 (4.6-10.6)	0.075
	At some moments	75	18.9 (12.0-28.5)		66	16.7 (9.9-26.6)		24	6.0 (2.6-13.3)	
	No	21	20.5 (12.8-31.3)		18	17.2 (9.5-29.1)		22	21.5 (7.5—48.2)	
Type of delivery										
	Vaginal or forceps	118	17.1 (14.6-19.9)		121	17.5 (13.8-21.9)		45	6.5 (3.5-12.0)	
	C-section without labor	168	21.4 (16.9-26.8)	0.055	138	17.7(14.5-21.5)	0.153	64	8.3 (6.5-10.5)	0.467
	C-section with spontaneous or induced labor	18	8.0 (2.6-22.1)		16	7.2 (2.2-21.6)		20	9.2 (5.3-15.5)	
Payment for hospitalization										
	Unified Health System/public	231	18.5 (14.4-23.4)	0.643	218	17.5 (14.2-21.5)	0.035	115	9.3 (6.9-12.3)	0.002
	Health insurance plan	57	15.9 (12.4-20.1)		49	13.7(10.5-1 7.7)		12	3.3 (1.8-6.1)	
	Out-of-pocket payment	15	17.7 (8.6-33.1)		7	7.6 (3.2-17.0)		2	2.8 (1.2-6.6)	
Weight at birth (g)										
	< 1,500	3	19.2 (3.2-62.9)	0.953	5	30.6 (6.7-72.8)	0.616	-	-	0.325
	1,500-2,499	26	19.0(11.3-30.1)		27	19.6 (8.5-38.5)		20	14.2 (3.7—41.2)	
	≥ 2,500	273	17.7(14.4-21.6)		241	15.7 (12.3-19.9)		109	7.1 (5.7-8.9)	
Gestational age (weeks)										
	< 37	35	19.6 (12.4-29.4)	0.127	38	21.3 (8.3-44.7)	0.260	27	16.2 (3.7-49.2)	0.351
	3 7—41	260	1 7.3 (12.7-23.2)		228	15.2 (10.6-21.4)		102	6.8 (5.6-8.2)	
	≥ 42	9	48.3 (28.2-69.0)		9	48.3 (28.2-69.0)		-	-	
Newborn admitted to an intensive care unit										
	Yes	14	13.1 (4.1-34.9)	0.503	21	19.9 (12.3-30.4)	0.397	42	41.8 (14.4-75.4)	< 0.001
	No	290	18.2 (15.2-21.8)		253	16.0 (13.3-19.2)		87	5.5 (4.4-6.9)	

a Total numbers range due to missing data in some category of the variable

b Symptoms of depression: score ³ 10 at the Edinburgh Postnatal Depression Scale (EPDS).

c Symptoms of anxiety: score ³ 10 at the Generalized Anxiety Disorder 7 (GAD 7).

d Symptoms of post-traumatic disorder associated to childbirth: score > 28 at the City Birth trauma Scale (City BiTS).

Maternal age under 20 was significantly higher among women with symptoms of depression compared to those aged 35 or older (p = 0.019). Adolescents also showed higher rates of anxiety compared to women aged 20 or older (p = 0.003). Skin color/race was a significant differentiating factor only for women with anxiety symptoms. Those identifying as black and brown had higher prevalences of anxiety (19.1 and 18.6%, respectively) compared to white women (10.2%).

The prevalence of severe maternal morbidity and hospitalization covered by SUS (compared to private insurance and out-of-pocket payment) was significantly higher among women with anxiety symptoms (p = 0.024 and 0.035, respectively) and PTSD symptoms (p = 0.008 and 0.002, respectively).

Data from Table 3 show that women with PTSD symptoms appeared to exhibit a greater level of vulnerability, with significantly higher prevalence rates in eight out of the 16 variables analyzed. In addition to the previously mentioned variables (education level, history of mental disorders, severe maternal morbidity, and delivery in the public sector), higher prevalence of PTSD was observed among multiparous women compared to primiparous women, those with one or two previous children, a history of abortion or stillbirth, twin pregnancies, and those whose babies were admitted to a neonatal ICU. However, it is worth noting that due to the small number of twin pregnancies and neonatal ICU admissions, the prevalences showed low precision in the estimates.

**Figure 1:**
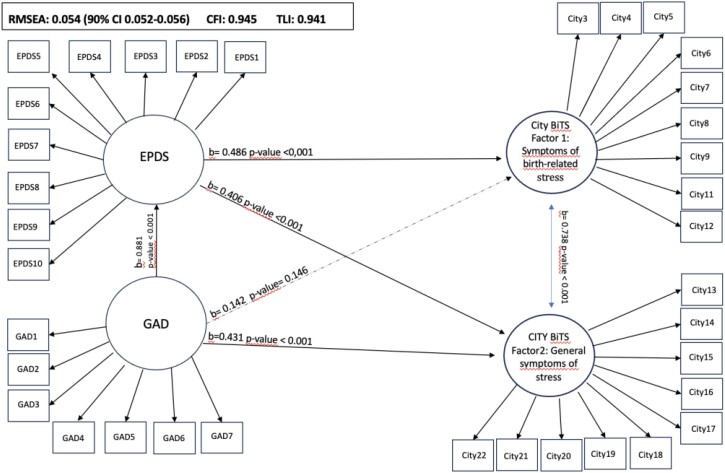
Theoretical model of the co-occurrence of symptoms of depression, anxiety, and birth-related post-traumatic stress disorder. *Nascer no Brasil* II study. Rio de Janeiro, 2021–2023.

RMSEA: *root mean square error approximation*; CFI: *comparative fit index*; TLI: Tucker-Lewis *index*; EPDS: Edinburgh Postnatal Depression Scale; GAD7: Generalized Anxiety Disorder 7; CITY BiTS: City Birth Trauma Scale.

Figure 1 shows that the theoretical model of the association between the presence of depression, anxiety, and PTSD symptoms achieved adequate fit indices (RMSEA = 0.054; 90%CI 0.052–0.056), CFI = 0.945, and TLI = 0.941. The structural equation model revealed a direct and significant effect between the presence of depression symptoms and anxiety symptoms (b = 0.881; p < 0.001) and PTSD symptoms (factor 1: b = 0.486; p < 0.001; factor 2: b = 0.40; p < 0.001), as well as between anxiety symptoms and factor 2 of the PTSD scale (b = 0.431; p < 0.001). However, the path of association between GAD-7 and factor 1 of the City BiTS was not significant (b = 0.142; p = 0.146). Additionally, all latent variables of the model had items with factor loadings greater than 0.5 (Table 4).

**Table 4:** Standardized coefficient, standard error, and p-value of the theoretical model of the association between symptoms of depression, anxiety, and birth-related post-traumatic stress disorder. *Nascer no Brasil* II study. Rio de Janeiro, 2021–2023.

Adjusted model		RMSEA	0.054 (IC90% 0.052–0.056)	
		CFI	0.945	
		TLI	0.941	
**Latent Variable**		**Standardized coefficient**	**Standard error**	**P-value**
City BiTS (factor 1)				
	City 3	0.574	0.046	< 0.001
	City 4	0.665	0.036	< 0.001
	City 5	0.875	0.022	< 0.001
	City 6	0.758	0.042	< 0.001
	City 7	0.674	0.033	< 0.001
	City 8	0.804	0.027	< 0.001
	City 9	0.873	0.021	< 0.001
	City 11	0.879	0.027	< 0.001
	City 12	0.584	0.056	< 0.001
City BiTS (factor 2)				
	City 13	0.788	0.025	< 0.001
	City 14	0.799	0.023	< 0.001
	City 15	0.810	0.019	< 0.001
	City 16	0.779	0.019	< 0.001
	City 17	0.807	0.017	< 0.001
	City 18	0.816	0.020	< 0.001
	City 19	0.744	0.021	< 0.001
	City 20	0.774	0.774	< 0.001
	City 21	0.829	0.015	< 0.001
	City 22	0.798	0.019	< 0.001
Post-partum depression (EPDS)				
	EPDS1	0.738	0.024	< 0.001
	EPDS2	0.692	0.024	< 0.001
	EPDS3	0.798	0.018	< 0.001
	EPDS4	0.795	0.017	< 0.001
	EPDS5	0.821	0.024	< 0.001
	EPDS6	0.579	0.024	< 0.001
	EPDS7	0.731	0.025	< 0.001
	EDPS8	0.873	0.014	< 0.001
	EPDS9	0.869	0.017	< 0.001
	EPDS10	0.804	0.031	< 0.001
Anxiety (GAD7)				
	GAD1	0.855	0.014	< 0.001
	GAD2	0.765	0.020	< 0.001
	GAD3	0.823	0.015	< 0.001
	GAD4	0.782	0.017	< 0.001
	GAD5	0.675	0.029	< 0.001
	GAD6	0.830	0.017	< 0.001
	GAD7	0.827	0.020	< 0.001
	City BiTS factor 1 and factor 2	0.738	0.738	< 0.001
**Direct effect**		**Standardized coefficient**	**Standard error**	**P-value**
City BiTS factor 1				
	EPDS	0.486	0.103	< 0.001
	GAD7	0.142	0.103	0.167
City BiTS factor 1				
	EPDS	0.406	0.065	< 0.001
	GAD7	0.025	0.060	< 0.001
EPDS				
	GAD7	0.881	0.012	< 0.001

RMSEA: *root mean square error approximation*.

CFI: *comparative fit index*.

TLI: Tucker-Lewis *index*.

EPDS: Edinburgh Postnatal Depression Scale.

GAD7: Generalized Anxiety Disorder 7.

CITY BiTS: City Birth Trauma Scale.

## DISCUSSION

The results of this article confirmed the high prevalence of depression and anxiety symptoms in the postpartum period, with a lower proportion of PTSD symptoms associated with childbirth. Additionally, comorbidity and triple morbidity were observed. The structural equation model revealed a direct and significant effect between the presence of depression symptoms and anxiety and PTSD symptoms, as well as between anxiety symptoms and factor 2 of the PTSD scale. The simultaneous occurrence of depression, anxiety, and PTSD symptoms in 3.7% (95%CI 3.0–4.6) supported the theoretical model. These results are consistent with the literature regarding the prevalence of mental disorders in the postpartum period^7,9^, as well as the presence of comorbidity and multimorbidity^14,32^.

Regarding the presence of comorbidity, there is evidence that the prevalence of postpartum anxiety and depression ranges from 10 to 20% in middle- and low-income countries^33^. A study conducted with postpartum women in Ethiopia also found an association between depression and anxiety (14.5%) six weeks after childbirth. However, compared to this research, other comorbidities showed higher prevalences, with 9.3% of anxiety and PTSD, 9.4% of depression and PTSD, and 7.4% of multimorbidity^34^.

Similar results were presented by Howard et al., who, using various analytical approaches, observed that postpartum depression, anxiety, and PTSD are closely related and often occur simultaneously. Postpartum depression scores increased linearly with anxiety and PTSD scores.

This study also confirms the greater social vulnerability among postpartum women with mental health problems. Research conducted in Brazil and other countries^11,16,18^ reaffirms that low socioeconomic status, a history of mental disorders, living without a partner, and having an unplanned pregnancy, among others, are associated with a higher prevalence of depression, anxiety, and stress symptoms in the postpartum period.

These results highlight the importance of investigating mental health issues during the perinatal period, given their high prevalence, both individually and in combination. Routine screening during pregnancy and the postpartum period has been adopted in some countries with the aim of providing early intervention and reducing maternal suffering and its repercussions on the health of both the mother and the baby. The American College of Obstetricians and Gynecologists recommends that all obstetric care providers conduct a comprehensive emotional well-being assessment (including screening for postpartum depression and anxiety using a validated instrument) during the postpartum visit^36^. The WHO recommends a care package with a step-by-step approach, including interventions for promotion and prevention, as well as the identification and treatment of mental health conditions^37^.

In Brazil, there are still no protocols for investigating perinatal mental disorders, making the problem underreported and underdiagnosed. Although some healthcare units have already implemented depression screening during pregnancy and the postpartum period, it is important that anxiety disorders and PTSD also be investigated, as comorbidity and multimorbidity are frequent events that share the same risk factors and affect women with greater social disadvantages, as demonstrated in the results of this study. Additionally, it is important for healthcare professionals to be aware of the potential complexity of multimorbidity occurrence.

Although this study presented results consistent with those of other studies, some methodological limitations may have influenced the results. One possible limitation is inherent to phone-based surveys, which are subject to missed calls, incorrect or non-existent phone numbers, and refusal to provide information over the phone, leading to a response rate of 64%. In any case, some statistical strategies were employed to address the non-response issue. Furthermore, it should be noted that studies collecting psychosocial information are more prone to social acceptability phenomena and stigma, as they address sensitive topics. As a result, participants tend to be less truthful in their responses in an attempt to avoid judgment from the interviewer or to meet social expectations, which may lead to an underestimation of the prevalence.

On the other hand, it is important to highlight that this is an innovative study, the first to investigate the prevalence of birth-related PTSD and the co-occurrence of depression and anxiety symptoms in a cohort of Brazilian women. Additionally, the data collection through various sources at baseline (interviews, prenatal cards, maternal and newborn records, and ultrasound results) addressed risk factors across multiple domains, allowing for the testing of various associations with the mental disorders investigated in the follow-up.

## CONCLUSION

This study highlights that the postpartum period is critical for the diagnosis of mental disorders and can involve complex conditions in which symptoms of depression, anxiety, and stress overlap. It is important that healthcare professionals are aware of the occurrence and co-occurrence of these disorders, as well as the potential consequences for the health of the woman and the newborn. Socioeconomic inequalities and healthcare system barriers may exacerbate the cycle of vulnerability and mental health issues. Recognizing and addressing these factors are essential for the effective prevention and treatment of perinatal mental disorders.

## Data Availability

The datasets generated and/or analyzed during the study are available from the corresponding author upon request.
