# Supplementary material for: Mental disorders in the Postpartum Period in Rio de Janeiro 2021–2023: Nascer no Brasil II Study
Source: Rev Saude Publica. 2025 Oct 20;59(Suppl 1):e8s. doi: 10.11606/s1518-8787.2025059006527 (PMC12539638; doi:10.11606/s1518-8787.2025059006527)
Supplement: Translation

# Translation: Transtornos mentais no pós-parto no Rio de Janeiro 2021–2023: Pesquisa Nascer no Brasil II

**Authors:** Mariza Miranda Theme-Filha, Marcia Leonardi Baldisserotto, Ana Claudia Santos Amaral, Maria Pappaterra Bastos, Arthur Orlando Correa Schilithz, Silvana Granado Nogueira da Gama, Karina de Cássia Caetano, Maria do Carmo Leal

**Affiliations:** I Fundação Oswaldo Cruz. Escola Nacional de Saúde Pública Sérgio Arouca. Departamento de Epidemiologia e Métodos Quantitativos em Saúde. Rio de Janeiro, RJ, Brasil; II Universidade Federal do Rio de Janeiro. Instituto de Psicologia. Departamento de Psicometria. Rio de Janeiro, RJ, Brasil; III Fundação Oswaldo Cruz. Instituto Nacional de Infectologia Evandro Chagas. Rio de Janeiro, RJ, Brasil

**Keywords:** Transtornos Mentais, Período Pós-Parto, Depressão, Ansiedade, Transtornos de Estresse Pós-Traumáticos

## Abstract

**OBJETIVO:**

Analisar a prevalência e a inter-relação entre os sintomas de depressão, ansiedade e estresse pós-traumático associado ao parto.

**MÉTODOS:**

Foram analisados os dados de uma coorte de puérperas da Pesquisa Nascer no Brasil II, representativa dos nascimentos ocorridos no estado do Rio de Janeiro entre 2021–2023, entrevistadas no pós-parto imediato (face a face) e dois meses após o nascimento (por telefone). Foram incluídas as mulheres que responderam a todas as perguntas das escalas Edinburgh Postnatal Depression Scale, Generalized Anxiety Disorder 7 e City Birth Trauma Scale e excluídas as gestações que terminaram em aborto ou natimorto, totalizando 1.752 puérperas. Para testar a homogeneidade das proporções, foi realizado o teste χ^2^ considerando significativo valores de p inferiores a 5%. A análise das inter-relações entre os três sintomas foi conduzida por meio de modelagem de equação estrutural (MEE), utilizando-se o estimador de média e variância dos mínimos quadrados ponderados e a parametrização theta.

**RESULTADOS:**

A prevalência de sintomas de depressão, ansiedade e estresse pós-traumático associado ao nascimento foi, respectivamente, 17,9, 16,3 e 7,7%. A presença de pontuação positiva em pelo menos uma escala foi de 24,6%, e a ocorrência simultânea de duas ou três comorbidades foi, respectivamente, 12 e 3,7%. A baixa escolaridade e transtorno mental prévio apresentaram prevalência significativamente mais elevada nos três transtornos analisados. MEE revelou associação significativa e positiva entre as três escalas, e todas as variáveis latentes do modelo apresentaram itens com cargas fatoriais superiores a 0,5.

**CONCLUSÃO:**

O pós-parto é crítico para o diagnóstico de transtornos mentais e pode cursar com quadros complexos em que sintomas de depressão, ansiedade e estresse se sobrepõem. É importante que os profissionais de saúde estejam cientes da ocorrência e coocorrência desses transtornos, bem como das possíveis consequências na saúde da mulher e na do recém-nascido.

## INTRODUÇÃO

O nascimento de um bebê pode ser uma das experiências mais gratificantes na vida de uma mulher, mas também é o período de maior vulnerabilidade, por causa das grandes transformações físicas e emocionais que ocorrem durante a gravidez e o pós-parto. Mesmo sem complicações, esse período é potencialmente estressante, aumentando o risco de desenvolver sintomas de transtornos mentais^1,2^.

Globalmente, os transtornos mentais maternos são considerados um grande desafio para a saúde pública. Em 2012, a Organização Mundial da Saúde (OMS) identificou a saúde mental perinatal, na gravidez e até um ano pós-parto como uma prioridade mundial^3^, e a melhora da qualidade da saúde materna faz parte dos Objetivos de Desenvolvimento do Milênio e dos Objetivos de Desenvolvimento Sustentável^4^. Problemas de saúde mental nesse período se manifestam, principalmente, com sintomas de depressão, ansiedade e estresse pós-traumático associado ao parto. A prevalência desses agravos varia com o momento do ciclo gravídico-puerperal em que foram aferidos, com os métodos de aferição (autorreferidos ou entrevista clínica) e com o contexto socioeconômico e cultural da população estudada^5^.

Os sintomas de depressão pós-parto são os mais frequentes, com prevalência média de 15,5% entre os países de alta renda^6^ e atingindo valores acima de 25% nos países de média e baixa renda^7^. No Brasil, dados nacionais revelam que 26,3% das mulheres apresentam sintomas de depressão até 12 meses após o nascimento do bebê^8^.

Os sintomas de ansiedade apresentam prevalência similar aos da depressão. Cerca de 20% das mulheres relatam sintomas de ansiedade no período perinatal, sendo mais frequentes durante a gravidez, se comparada com o pós-parto^9^. Revisão sistemática incluindo estudos em diversos contextos socioeconômicos revelou prevalência média de sintomas autorreferidos de 29,2% no pré-natal e 24,4% pós-natal, entretanto os casos diagnosticados clinicamente variam de 8,1% no pré-natal a 16% no pós-parto^7,10^.

Os sintomas do transtorno de estresse pós-traumático (TEPT) associado ao nascimento estão relacionados às experiências e percepções negativas do parto pela mulher, que ela percebe como ameaça para sua vida ou para a vida do bebê^11^. O TEPT afeta entre 3 e 6% das mães, e naquelas que apresentaram complicações obstétricas pode atingir 19%^12^. Assim como os sintomas de depressão e ansiedade, a prevalência varia de acordo com o perfil socioeconômico, com valores médios acima de 8% em países de média e baixa renda^7^.

As puérperas com sintomas de depressão, ansiedade e estresse pós-traumático associado ao parto compartilham vários fatores de risco, além de sintomas sobrepostos que podem se influenciar de várias maneiras, apresentando inter-relações entre eles^13–15^. Os principais fatores de risco incluem diversos domínios, como socioeconômico, biológico, psicossocial e obstétrico, com destaque para os transtornos mentais prévios e no período perinatal, complicações durante o parto e nascimento, experiência negativa do parto e falta de apoio social^16–18^.

Uma vez que as mulheres no período pós-parto podem apresentar sintomas complexos que se sobrepõem, é importante conhecer a prevalência e a ocorrência simultânea desses agravos, sabendo que a saúde mental materna tem grande impacto na vinculação dos pais com o bebê, no desenvolvimento infantil e no funcionamento familiar. Nossa hipótese é que puérperas com triagem positiva para sintomas de depressão também apresentam triagem positiva para sintomas de ansiedade e estresse pós-traumático no pós-parto.

No Brasil, estudos de base populacional sobre a prevalência dos transtornos mentais maternos no pós-parto são escassos. Particularmente, não há estudos sobre a inter-relação entre sintomas de depressão, ansiedade e estresse pós-traumático associado ao parto. Este estudo teve como objetivo preencher essa lacuna analisando uma coorte de puérperas brasileiras entrevistadas no pós-parto imediato e dois meses após o nascimento, com base nos dados da Pesquisa Nascer no Brasil II (NBII), pesquisa nacional sobre aborto, parto e nascimento.

## MÉTODOS

A pesquisa NBII utiliza uma amostra probabilística representativa dos nascimentos hospitalares no Brasil, com aproximadamente 20 mil puérperas internadas para o parto (de nascido vivo ou nascido morto) ou assistência ao abortamento, com coleta de dados no período de 2021 a 2024.

A amostra foi realizada com dois estágios de seleção, correspondendo às maternidades e puérperas, e estratificada segundo macrorregião do país (norte, nordeste, sudeste, sul, centro-oeste); tipo de hospital (público/misto/privado); localização (capital e municípios localizados em região metropolitana e demais municípios; e número de nascimentos vivos/ano (100–499 NV/ano, ≥ 500 NV/ano). Além da entrevista face a face com as puérperas na maternidade, em média seis horas após o nascimento/aborto, a pesquisa incluiu a coleta de dados de prontuário, dos cartões de pré-natal e de resultados de exame de ultrassonografia. Ela aborda vários aspectos socioeconômicos, comportamentais, atenção pré-natal, admissão na maternidade, trabalho de parto, parto e pós-parto imediato, além das circunstâncias do aborto e procedimentos realizados. Duas entrevistas de seguimento foram realizadas por ligação telefônica aos dois e quatro meses após o parto/aborto com as mulheres que participaram da linha de base na maternidade e abordaram a presença de morbidade materna e neonatal, uso de serviços de saúde pós-natais, aleitamento materno, satisfação com o atendimento ao parto e aborto, sintomas de depressão, ansiedade e de TEPT associado ao parto, violência obstétrica, vínculo mãe-bebê e discriminação no dia a dia. Mais detalhes sobre o método da pesquisa NBII se encontram disponíveis em Leal et al.^19^ e Theme Filha et al.^20^.

Para o estado do Rio de Janeiro, foi delineada uma amostra representativa dos nascimentos ocorridos entre 2021–2023, totalizando 1.923 puérperas. Na presente análise, utilizaram-se os dados da linha de base e do primeiro seguimento por telefone, excluindo os abortos (n = 161) e natimortos (n = 10), totalizando 1.752 puérperas. Como não foi possível o seguimento de todas as mulheres da linha de base (taxa de resposta de 64%), foi ajustado um modelo de regressão logística para estimar a probabilidade de cada mulher responder à entrevista telefônica, mediante um conjunto de variáveis que diferenciaram as respondentes das não respondentes, por meio de escore de propensão. Esse procedimento teve o intuito de compensar a tendência de as mulheres com certas características não responderem à entrevista telefônica, supondo que teriam fornecido, em média, respostas semelhantes das respondentes em cada estrato e categoria de ajustamento.

Foram testadas variáveis relacionadas ao local do parto, sociodemográficas e obstétricas. O modelo logístico mais parcimonioso incluiu as variáveis estatisticamente significativas (p < 0,05), que diferenciaram respondentes de não respondentes. As não respondentes tinham menos de 16 anos de estudo, tiveram o parto em hospitais públicos (em comparação com os hospitais privados e mistos) e naqueles com menos de 500 partos/ano. As principais causas de não resposta foram telefone errado e não atender às ligações. As recusas foram inferiores a 5%. Por se tratar de uma amostra complexa, foi utilizada, além dos pesos, a correção do efeito do desenho.

Na análise das prevalências dos sintomas de depressão, ansiedade e TEPT associado ao parto, foram incluídas as mulheres que responderam a todas as perguntas das escalas (taxa de resposta acima de 96% para as três escalas) e seus respectivos intervalos de confiança de 95%, considerando os pontos de corte válidos para o Brasil. Para testar a homogeneidade das proporções, foi realizado o teste χ^2^ considerando significativo os valores de p inferiores a 5%.

Para aferição dos sintomas de depressão, ansiedade e TEPT associado ao parto, foram utilizadas, respectivamente, as escalas Edinburgh Postnatal Depression Scale (EPDS), Generalized Anxiety Disorder 7 (GAD7) e City Birth Trauma Scale (City BiTS) dois meses após o parto.

A EPDS^21^ contém 10 itens que avaliam sintomatologia depressiva perinatal, com pontuação total variando de 0 a 30. A escala foi validada para uso por entrevista telefônica no Brasil, e o ponto de corte ≥ 10 é indicativo de possível depressão pós-parto^22^.

A escala GAD7^23^ é composta de sete itens, que aferem sintomas de ansiedade, com quatro opções de resposta de acordo com uma escala Likert variando de 0 (nenhuma vez) a 3 (quase todos os dias), com escore máximo de 21 pontos. Foi traduzida para o português brasileiro^24^, e o ponto de corte ≥ 10 é sugestivo da presença de sintomas de ansiedade de intensidade moderada/severa.

A City BiTS^25^, em sua versão completa, contém 29 itens que aferem o TEPT associado ao nascimento de acordo com as definições da quinta edição do Manual Diagnóstico e Estatístico de Transtornos Mentais^26^, abordando as dimensões intrusão, evitação e alteração de humor. Neste estudo, nas análises descritivas e bivariadas foram utilizados os 20 itens que avaliam sintomas de estresse traumático. A escala é válida para uso no Brasil^27^, e o ponto de corte > 28 indica escore positivo para o contexto brasileiro^28^. Nas análises de modelagem de equação estrutural foi utilizado o modelo fatorial proposto pelos autores^25^ composto de dois fatores, sendo o fator 1 constituído de nove itens relativos aos sintomas de TEPT associado ao nascimento, e o fator 2, de 10 itens relativos aos sintomas gerais de estresse.

Foram analisadas as variáveis sociodemográficas (idade, escolaridade, raça/cor, situação conjugal), antecedentes obstétricos (paridade, aborto e natimorto prévio), histórico de transtorno mental prévio (relato de pelo menos um dos seguintes transtornos: ansiedade, depressão, TEPT, esquizofrenia, transtorno obsessivo-compulsivo, transtorno bipolar), momento em que ocorreu a gestação (você sente que a sua gravidez aconteceu no momento certo; não bem no momento certo; no momento errado), tipo de gestação (única ou gemelar), morbidade materna grave, tipo de parto (vaginal/fórceps ou cesariana com ou sem trabalho de parto), a presença de acompanhante durante a internação, forma de pagamento do parto (Sistema Único de Saúde — SUS/plano de saúde/desembolso direto) e condições de nascimento do bebê (peso e idade gestacional ao nascer, internação em unidade de terapia intensiva neonatal). A variável morbidade materna grave considerou a presença de pelo menos um critério de condição potencialmente ameaçadora da vida ou morbidade materna grave, conforme definido pela OMS^29^.

A análise das inter-relações entre sintomas de depressão, ansiedade e TEPT associado ao parto foi conduzida por meio de modelagem de equação estrutural (MEE). Foram utilizados o estimador de média e variância dos mínimos quadrados ponderados (WLSMV) e a parametrização theta. Dessas análises, foram obtidos os coeficientes de regressão padronizados estimados, seus respectivos erros padrão e valores p associados para averiguação da associação entre as variáveis do modelo. Esses parâmetros foram obtidos utilizando a estratégia *bootstrap* com cinco mil amostras. Foi adotado o valor p inferior a 0,05 para indicar significância estatística.

A adequação do modelo foi avaliada pelos índices de ajuste *comparative fit index* (CFI), Tucker-Lewis *index* (TLI) e *root mean square error of approximation* (RMSEA). Adotaram-se os valores de CFI e TLI > 0,95 e RMSEA < 0,06 para indicar o modelo adequado. Para o RMSEA, foi adotado o intervalo de confiança de 90% (IC90%), e consideraram-se adequados um limite inferior próximo de 0 e um superior até 0,08^30,31^. Todas as análises estatísticas foram realizadas mediante o Statistical Package for the Social Sciences R versão 3.5.1 e o MPlus versão 8.

### Aspectos Éticos

A pesquisa NBII foi aprovada pela Comissão Nacional de Ética em Pesquisa, de acordo com o parecer número 3.909.299, em 11 de março de 2020.

## RESULTADOS

A Tabela 1 destaca que a maioria das entrevistadas estava na faixa etária de 20 a 34 anos (71,3%; intervalo de confiança de 95% — IC95% 68,3–74,1), relatou cor da pele/raça parda (50,2%; IC95% 45,6–54,9), de nove a 15 anos de estudo (68,7%; IC95% 63,5–73,4) e viver com companheiro (83,1%; IC95% 80,4–85,6). Em relação às variáveis obstétricas, 46,5% (IC95% 42,7–50,3) tiveram um ou dois partos anteriores, e a história prévia de aborto/natimorto foi citada por 20,6% (IC95% 16,8–24,9). Destaca-se a elevada proporção de algum transtorno mental prévio (12,8%; IC95% 10,8–15,1) e de gravidez não planejada (35,4%). A gravidez ocorreu não bem no momento certo para 26,6% (IC95% 21,4–32,5), e 8,8% (IC95% 6,8–11,2) delas consideraram a gravidez no momento errado. Dados dos prontuários registraram apenas 12 casos de gestação gemelar, e 14,2% (IC95% 11,1–18,1) das puérperas evoluíram com complicações graves durante o trabalho de parto ou parto. A presença de acompanhante durante toda a internação foi relatada por 70,7% (IC95% 60,3–79,3), e apenas 5,9% (IC95% 2,5–13,4) não teve acompanhante em momento nenhum. A cesariana eletiva sem trabalho de parto foi a via de nascimento em 46,6% (IC95% 33,9–59,8) das gestantes, e 70% das internações foram pagas pelo SUS. Os recém-nascidos apresentaram, em sua grande maioria, boas condições de saúde, com registro de baixo peso e prematuridade em torno de 10%, e 6,2% (IC95% 4,0–9,6) deles foram internados em unidades intermediárias/unidades de terapia intensiva (UTI) neonatal.

**Tabela 1:** Descrição da amostra. Pesquisa Nascer no Brasil II. Rio de Janeiro, 2021–2023.

Variáveis		Amostra (n = 1.752)	
		n	% (IC95%)
	Faixa etária (anos)		
	< 20	171	9,8 (7,3–12,9)
	20–34	1.249	71,3 (68,3–74,1)
	≥ 35	333	19,0 (15,9–22,5)
Cor da pele/raça			
	Branca	512	29,2 (24,9–33,9)
	Preta	360	20,6 (16,9–24,8)
	Parda	880	50,2 (45,6–54,9)
Anos de estudo			
	≤ 8	254	14,5 (11,2–18,7)
	9–15	1.204	68,7 (63,5–73,4)
	≥ 16	294	16,8 (12,7–21,9)
Situação conjugal			
	Vive com companheiro(a)	1.454	83,1 (80,4–85,6)
	Não vive com companheiro(a)	295	16,9 (14,4–19,6)
Paridade			
	Primípara	747	42,6 (37,7–47,7)
	Um ou dois partos anteriores	815	46,5 (42,7–50,3)
	Três ou mais partos anteriores	190	10,8 (9,3–12,6)
Aborto/natimorto prévio			
	Sim	360	20,6 (16,8–24,9)
	Não	1.392	79,4 (75,1–83,2)
Transtorno mental prévio			
	Sim	224	12,8 (10,8–15,1)
	Não	1.528	87,2 (84,9–89,2)
Momento em que ocorreu a gravidez			
	No momento certo	1.036	64,6 (60,0–69,0)
	Não bem no momento certo	426	26,6 (21,4–32,5)
	No momento errado	141	8,8 (6,8–11,2)
Tipo de gestação			
	Única	1.740	99,3 (98,5–99,7)
	Gemelar	12	0,7 (0,3–1,5)
Morbidade materna grave			
	Sim	250	14,2 (11,1–18,1)
	Não	1.502	85,8 (81,9–88,9)
Acompanhante no trabalho de parto/parto			
	Todos os momentos	1.239	70,7 (60,3–79,3)
	Em alguns momentos	409	23,4 (17,9–29,9)
	Em nenhum momento	104	5,9 (2,5–13,4)
Tipo de parto			
	Vaginal ou fórceps	703	40,1 (33,6–47,1)
	Cesariana sem trabalho de parto	816	46,6 (33,9–59,8)
	Cesariana com trabalho de parto espontâneo ou induzido	232	13,3 (7,5–22,4)
Pagamento da internação			
	Sistema Único de Saúde/público	1.288	73,6 (61,1–83,2)
	Plano de saúde	370	21,2 (12,3–33,8)
	Desembolso direto	92	5,3 (2,3–11,8)
Peso ao nascer			
	< 1.500 g	16	0,9 (0,3–2,5)
	1.500–2.499	142	8,1 (6,3–10,5)
	≥ 2.500 g	1.591	90,9 (88,4–93,0)
Idade gestacional ao nascer (semanas)			
	< 37	183	10,4 (7,9–13,8)
	37 a 41	1.549	88,4 (84,6–91,4)
	≥ 42	20	1,1 (0,3–4,0)
Recém-nascido admitido em unidade de terapia intensiva neonatal			
	Sim	109	6,2 (4,0–9,6)
	Não	1.643	93,8 (90,4–96,0)

IC95%: intervalo de confiança de 95.

A Tabela 2 revela que a prevalência de sintomas de depressão, ansiedade e TEPT associado ao nascimento foi, respectivamente, 17,9 (IC95% 14,6–21,9), 16,3 (IC95% 13,7–19,2) e 7,7% (IC95% 5,5–10,5). A presença de pontuação positiva em pelo menos uma escala foi de 24,6% (IC95% 21,6–27,9), e a ocorrência simultânea de duas ou três comorbidades foi, respectivamente, 12 (IC95% 10,3–13,9) e 3,7% (IC95% 3,0–4,6). A prevalência de sintomas de depressão com ansiedade foi de 10,5% (IC95% 8,8–12,5) e com TEPT de 4,2% (IC95% 3,6–5,1). A coocorrência de sintomas de ansiedade e TEPT alcançou 4,7% (IC95% 3,9–5,6).

**Tabela 2:** Prevalência de sintomas de depressão, ansiedade, estresse pós-traumático associado ao parto e comorbidades. Pesquisa Nascer no Brasil II. Rio de Janeiro, 2021–2023^a^.

Variáveis	n	% (IC95%)
Sintomas de depressão	304	17,9 (14,6–21,9)
Sintomas de ansiedade	275	16,3 (13,7–19,2)
Sintomas de estresse pós-traumático associado ao parto	129	7,7 (5,5–10,5)
Pelo menos uma escala positiva	432	24,6 (21,6–27,9)
Duas escalas positivas	210	12,0 (10,3–13,9)
Três escalas positivas	65	3,7 (3,0–4,6)
Sintomas de depressão e ansiedade	184	10,5 (8,8–12,5)
Sintomas de depressão e estresse pós-traumático associado ao parto	74	4,2 3,6–5,1)
Sintomas de ansiedade e estresse pós-traumático associado ao parto	82	4,7 (3,9–5,6)

a Sintomas de depressão: escore ≥ 10 na Edinburgh Postnatal Depression Scale (EPDS); sintomas de ansiedade: escore ≥ 10 na Generalized Anxiety Disorder 7 (GAD 7); sintomas de transtorno de estresse pós-traumático associado ao parto: escore > 28 na City Birth Trauma Scale (City BiTS).

IC95%: intervalo de confiança de 95.

Na Tabela 3, observa-se que puérperas com baixa escolaridade (menos de oito anos de estudo) e história de transtorno mental prévio apresentaram prevalências estatisticamente maiores nos três transtornos analisados em comparação às outras categorias dessas variáveis.

**Tabela 3:** Prevalência de sintomas de depressão, ansiedade e estresse pós-traumático associado ao parto segundo variáveis sociodemográficas, antecedentes clínicos e obstétricos, trabalho de parto/parto e condições de nascimento. Pesquisa Nascer no Brasil II. Rio de Janeiro, 2021–2023.

Variável		Síntomas de depressáo^b^ (n = 304)		Valor p	Síntomas de ansiedade^c^ (n = 275)		Valor p	Síntomas de TEPT^d^ (n = 129)		Valor p
		n^a^	% (IC95%)		n^a^	% (IC95%)		n^a^	% (IC95%)	
Iclacle materna (anos)										
	< 20	35	21,4 (17,2-26,3)	0,019	36	22,2 (16,3-29,5)	0,003	13	7,8 (4,9-12,4)	0,913
	20-34	235	19,5 (15,5-24,1)		211	1 7,5 (14,3-21,4)		90	7,5 (5,7-9,8)	
	≥ 35	33	10,3 (5,6-18,2)		27	8,3 (4,9-13,9)		26	8,1 (3,9-16,0)	
Cor da pele/raça										
	Branca	70	14,2 (10,7-1 8,6)	0,1 70	50	10,2 (5,8-1 7,3)	0,014	33	6,7 (4,0-11,0)	0,463
	Prêta	75	21,6 (15,7-28,9)		67	19,1 (15,9-22,8)		24	9,7 (6,0-15,2)	
	Parda	159	18,6 (13,5-25,1)		158	18,6 (15,8-21,7)		63	7,4 (4,9-11,1)	
Anos de estudo										
	< 8	60	25,0 (19,1-32,0)	0,001	64	26,9 (20,8-34,1)	< 0,001	32	13,3 (7,7-21,9)	0,004
	9-15	219	18,6 (14,1-24,3)		191	16,2 (13,2-1 9,8)		92	7,9 (5,8-1 0,6)	
	≥ 16	24	8,7 (5,7-13,0)		20	7,2 (4,4-11,5)		6	2,0 (0,6-6,0)	
Situaçâo conjugal										
	Vive com companheiro(a)	245	1 7,4 (14,3-21,1)	0,307	213	15,2 (12,7-1 8,0)	0,114	113	8,1 (5,2-12,4)	0,332
	Nâo vive com companheiro(a)	57	19,8 (14,1-27,1)		60	20,9 (14,1-30,0)		14	4,9 (2,4-9,9)	
Paridade										
	Primípara	126	1 7,6 (13,5-22,5)	0,315	105	14,6 (11,2-1 9,0)	0,169	36	5,0 (3,5-7,2)	0,030
	Um ou dois partos anteriores	133	16,7(11,4-23,8)		113	14,3 (7,3-26,0)		66	8,4 (6,1-11,6)	
	Très ou mais partos anteriores	45	24,7 (16,7-34,9)		57	31,2 (1 8,4-47,7)		27	14,9 (6,3-31,1)	
Aborto/natimorto previo										
	Sim	72	20,4 (14,5-27,9)	0,188	64	18,2 (13,5-24,1)	0,2 76	41	11,7 (6,8-1 9,4)	0,012
	Nao	232	1 7,3 (14,3-20,7)		211	15,7 (13,4-1 8,5)		88	6,6 (4,8-9,0)	
Transtorno mental previo										
	Sim	78	36,5 (26,2—48,2)	0,004	66	30,9 (1 9,0—45,9)	0,028	35	16,4 (12,2-21,7)	< 0,001
	Nao	225	15,2 (10,9-20,8)		209	14,1 (10,5-18,7)		94	6,4 (4,2-9,6)	
Momento em que ocorreu a gravidez										
	No momento certo	166	16,6 (13,2-20,6)	0,135	150	14,9 (12,4-1 7,8)	0,058	80	7,9 (5,8-1 0,8)	0,515
	Nao bem no momento certo	88	21,5 (16,8-2 7,2)		87	21,7 (18,0-25,8)		40	10,1 (3,9-23,5)	
	No momento errado	31	22,6 (12,7-36,9)		25	18,6 (10,3-31,4)		7	4,9 (1,8-12,5)	
Tipo de gestapáo										
	Unica	298	17,7 (14,3-21,7)	0,053	2 70	16,1 (13,6-19,1)	0,214	124	7,4 (5,2-1 0,4)	< 0,001
	Cemelar	6	45,8 (17,2-77,3)		4	33,2 (9,7-69,7)		6	45,8 (1 7,2-77,3)	
Morbidade materna grave										
	Sim	50	20,4 (14,3-28,2)	0,306	54	22,1 (1 5,8-29,9)	0,024	38	16,1 (7,5-31,2)	0,008
	Nao	253	17,5 (14,2-21,4)		220	15,3 (12,9-18,0)		91	6,3 (5,1-7,8)	
Acompanhante no trabalho de parto/parto										
	Todos os momentos	207	17,4 (14,8-20,3)	0,665	191	16,0 (13,7-18,7)	0,909	84	7,0 (4,6-1 0,6)	0,075
	Em alguns momentos	75	18,9 (12,0-28,5)		66	16,7(9,9-26,6)		24	6,0 (2,6-13,3)	
	Em nenhum momento	21	20,5 (12,8-31,3)		18	1 7,2 (9,5-29,1)		22	21,5 (7,5-48,2)	
Tipo de parto										
	Vaginal ou fórceps	118	17,1 (14,6-19,9)		121	17,5 (13,8-21,9)		45	6,5 (3,5-12,0)	
	Cesariana sem trabalho de parto	168	21,4 (16,9-26,8)	0,055	138	1 7,7 (14,5-21,5)	0,153	64	8,3 (6,5-10,5)	0,467
	Cesariana com trabalho de parto espontáneo ou induzido	18	8,0 (2,6-22,1)		16	7,2 (2,2-21,6)		20	9,2 (5,3-15,5)	
Pagamento da internapao										
	Sistema Unico de Saúde/público	231	18,5 (14,4-23,4)	0,643	218	1 7,5 (14,2-21,5)	0,035	115	9,3 (6,9-12,3)	0,002
	Plano de saúde	57	15,9 (12,4-20,1)		49	13,7(10,5-1 7,7)		12	3,3 (1,8-6,1)	
	Desembolso direto	15	1 7,7 (8,6-33,1)		7	7,6 (3,2-1 7,0)		2	2,8 (1,2-6,6)	
Peso ao nascer (g)										
	< 1.500	3	19,2 (3,2-62,9)	0,953	5	30,6 (6,7-72,8)	0,616	-	-	0,325
	1.500-2.499	26	19,0(11,3-30,1)		27	19,6 (8,5-38,5)		20	14,2 (3,7—41,2)	
	≥ 2.500	2 73	1 7,7 (14,4-21,6)		241	15,7 (12,3-1 9,9)		109	7,1 (5,7-8,9)	
Idade gestacional (semanas)										
	< 37	35	19,6 (12,4-29,4)	0,127	38	21,3 (8,3-44,7)	0,260	27	16,2 (3,7—49,2)	0,351
	3 7-41	260	1 7,3 (12,7-23,2)		228	15,2 (10,6-21,4)		102	6,8 (5,6-8,2)	
	≥ 42	9	48,3 (28,2-69,0)		9	48,3 (28,2-69,0)		-	-	
Recém-nascido admitido em unidade de terapia intensiva neonatal										
	Sim	14	13,1 (4,1-34,9)	0,503	21	19,9 (12,3-30,4)	0,397	42	41,8 (14,4-75,4)	< 0,001
	Nao	290	18,2 (15,2-21,8)		253	16,0 (13,3-1 9,2)		87	5,5 (4,4-6,9)	

a Os totais variam por causa de dados faltantes em alguma categoria da variável.

b Sintomas de depressão: escore ³ 10 na Edinburgh Postnatal Depression Scale (EPDS).

c Sintomas de ansiedade: escore ³ 10 na Generalized Anxiety Disorder 7 (GAD 7).

d Sintomas de transtorno de estresse pós-traumático associado ao parto: escore > 28 na City Birth trauma Scale (City BiTS).

A idade materna inferior a 20 anos foi significativamente mais elevada entre as mulheres com sintomas de depressão quando comparadas com as com 35 anos ou mais (p = 0,019). As adolescentes apresentaram também maiores proporções de ansiedade quando comparadas com as de 20 anos ou mais (p = 0,003). A cor da pele/raça diferenciou significativamente apenas as mulheres com sintomas de ansiedade. Aquelas com cor da pele/raça preta e parda apresentaram maiores prevalências de ansiedade (19,1 e 18,6%, respectivamente) quando comparadas com as de cor/raça branca (10,2%).

As prevalências de morbidade materna grave e de pagamento da internação pelo SUS (quando comparado com pagamento pelo plano de saúde e desembolso direto) foram estatisticamente mais elevadas nas mulheres com sintomas de ansiedade (p = 0,024 e 0,035, respectivamente) e sintomas de TEPT (p = 0,008 e 0,002, respectivamente).

Observa-se nos dados da Tabela 3 que as mulheres com sintomas de TEPT pareceram apresentar um quadro de maior vulnerabilidade, com prevalências significativamente mais elevadas em oito das 16 variáveis analisadas. Além das variáveis mencionadas anteriormente (escolaridade, transtorno mental prévio, morbidade materna grave e parto no setor público), as multíparas em relação às primíparas e com um ou dois filhos anteriores, com aborto/natimorto prévio, gestação gemelar e bebê internado em UTI neonatal apresentaram maiores prevalências de TEPT. Todavia, vale destacar que por causa do pequeno número de gestações gemelares e internação em UTI neonatal as prevalências apresentaram baixa precisão das estimativas.

A Figura 1 demonstra que o modelo teórico da associação entre a presença de sintomas de depressão, ansiedade e TEPT obteve índices de ajuste adequados (RMSEA = 0,054; IC90% 0,052–0,056), CFI = 0,945 e TLI = 0,941. O modelo de equações estruturais revelou um efeito direto e significativo entre a presença de sintomas de depressão e sintomas de ansiedade (b = 0,881; p < 0,001) e de TEPT (fator 1: b = 0,486; p < 0,001; fator 2: b = 0,40; p < 0,001) e dos sintomas de ansiedade com o fator 2 da escala de TEPT (b = 0,431; p < 0,001), porém o caminho de associação entre GAD-7 e o fator 1 da City BiTS não se mostrou significante (b = 0,142; p = 0,146). Adicionalmente, todas as variáveis latentes do modelo apresentaram itens com cargas fatoriais superiores a 0,5 (Tabela 4).

**Figura 1:** Modelo teórico da coocorrência de sintomas de depressão, ansiedade e estresse pós-traumático associado ao parto. Pesquisa Nascer no Brasil II. Rio de Janeiro, 2021–2023.

RMSEA: *root mean square error approximation*; CFI: *comparative fit index*; TLI: Tucker-Lewis *index;* EPDS: Edinburgh Postnatal Depression Scale; GAD7: Generalized Anxiety Disorder 7; CITY BiTS: City Birth Trauma Scale.

**Tabela 4:** Coeficiente padronizado, erro padrão e valor p do modelo teórico da associação entre sintomas de depressão, ansiedade e transtorno de estresse pós-traumático associado ao nascimento. Pesquisa Nascer no Brasil II. Rio de Janeiro, 2021–2023.

Modelo ajustado		RMSEA	0,054 (IC90% 0,052–0,056)	
		CFI	0,945	
		TLI	0,941	
**Variável latente**		**Coeficiente padronizado**	**Erro padrão**	**Valor p**
City BiTS (fator 1)				
	City 3	0,574	0,046	< 0,001
	City 4	0,665	0,036	< 0,001
	City 5	0,875	0,022	< 0,001
	City 6	0,758	0,042	< 0,001
	City 7	0,674	0,033	< 0,001
	City 8	0,804	0,027	< 0,001
	City 9	0,873	0,021	< 0,001
	City 11	0,879	0,027	< 0,001
	City 12	0,584	0,056	< 0,001
City BiTS (fator 2)				
	City 13	0,788	0,025	< 0,001
	City 14	0,799	0,023	< 0,001
	City 15	0,810	0,019	< 0,001
	City 16	0,779	0,019	< 0,001
	City 17	0,807	0,017	< 0,001
	City 18	0,816	0,020	< 0,001
	City 19	0,744	0,021	< 0,001
	City 20	0,774	0,774	< 0,001
	City 21	0,829	0,015	< 0,001
	City 22	0,798	0,019	< 0,001
Depressão pós-parto (EPDS)				
	EPDS1	0,738	0,024	< 0,001
	EPDS2	0,692	0,024	< 0,001
	EPDS3	0,798	0,018	< 0,001
	EPDS4	0,795	0,017	< 0,001
	EPDS5	0,821	0,024	< 0,001
	EPDS6	0,579	0,024	< 0,001
	EPDS7	0,731	0,025	< 0,001
	EDPS8	0,873	0,014	< 0,001
	EPDS9	0,869	0,017	< 0,001
	EPDS10	0,804	0,031	< 0,001
Ansiedade (GAD7)				
	GAD1	0,855	0,014	< 0,001
	GAD2	0,765	0,020	< 0,001
	GAD3	0,823	0,015	< 0,001
	GAD4	0,782	0,017	< 0,001
	GAD5	0,675	0,029	< 0,001
	GAD6	0,830	0,017	< 0,001
	GAD7	0,827	0,020	< 0,001
	City BiTS fator 1 e fator 2	0,738	0,738	< 0,001
Efeito direto		Coeficiente padronizado	Erro padrão	Valor p
City BiTS fator 1				
	EPDS	0,486	0,103	< 0,001
	GAD7	0,142	0,103	0,167
City BiTS fator 1				
	EPDS	0,406	0,065	< 0,001
	GAD7	0,025	0,060	< 0,001
EPDS				
	GAD7	0,881	0,012	< 0,001

RMSEA: *root mean square error approximation*.

CFI: *comparative fit index*.

TLI: Tucker-Lewis *index*.

EPDS: Edinburgh Postnatal Depression Scale.

GAD7: Generalized Anxiety Disorder 7.

CITY BiTS: City Birth Trauma Scale.

## DISCUSSÃO

Os resultados deste artigo confirmaram a elevada prevalência de sintomas de depressão e ansiedade no pós-parto, e em menor proporção de sintomas de TEPT associado ao nascimento. Adicionalmente, observou-se ocorrência de comorbidade e tripla morbidade. O modelo de equações estruturais revelou um efeito direto e significativo entre a presença de sintomas de depressão e sintomas de ansiedade e de TEPT, assim como dos sintomas de ansiedade com o fator 2 da escala de TEPT. A ocorrência simultânea de sintomas de depressão, ansiedade e TEPT de 3,7% (IC95% 3,0–4,6) corroborou com a proposta do modelo teórico. Esses resultados são consistentes com a literatura no que diz respeito à prevalência de transtornos mentais no pós-parto^7,9^, bem como à presença de comorbidade e multimorbidade^14,32^.

Em relação à presença de comorbidade, há evidências de que a prevalência de ansiedade e depressão pós-parto varia entre 10 e 20% nos países de média e baixa renda^33^. Estudo realizado com puérperas na Etiópia também encontrou associação de depressão e ansiedade (14,5%) seis semanas após o parto. Entretanto, comparativamente a esta pesquisa, as outras comorbidades apresentaram prevalências mais elevadas, sendo 9,3% de ansiedade e TEPT, 9,4% de depressão e TEPTe 7,4% de multimorbidade^34^.

Resultados semelhantes foram apresentados por Howard et al.^35^, que, utilizando várias abordagens analíticas, observaram que depressão, ansiedade e TEPT no pós-parto estão intimamente ligados e com frequência ocorrem simultaneamente. Os escores de depressão pós-parto aumentaram linearmente com as pontuações de ansiedade e de TEPT.

Este estudo também confirma a situação de maior vulnerabilidade social entre as puérperas com problemas de saúde mental no pós-parto. Pesquisas realizadas no Brasil^8^ e em outros países^11,16,18^ ratificam que baixo *status* socioeconômico, histórico anterior de transtornos mentais, viver sem companheiro e ter gravidez não planejada, entre outros se associam à maior prevalência de sintomas de depressão, ansiedade e estresse no pós-parto.

Esses resultados apontam para a importância da investigação dos problemas de saúde mental no período perinatal, ante sua elevada prevalência, isoladamente ou em combinação. A triagem de rotina durante a gravidez e o pós-parto tem sido adotada em alguns países com os objetivos de fornecer intervenção precoce e reduzir o sofrimento materno e suas repercussões na saúde da mulher e do bebê. O Colégio Americano de Obstetras e Ginecologistas recomenda que todos os prestadores de cuidados obstétricos realizem uma avaliação completa do bem-estar emocional (incluindo triagem para depressão e ansiedade pós-parto com um instrumento validado) durante a consulta puerperal^36^. A OMS recomenda um pacote de cuidados com uma abordagem passo a passo, incluindo intervenções para promoção e prevenção, identificação e tratamento de condições de saúde mental^37^.

No Brasil, ainda não se dispõem protocolos de investigação para os transtornos mentais perinatais, tornando o problema subnotificado e subdiagnosticado. Embora algumas unidades de saúde já tenham instituído o rastreio da depressão durante a gravidez e o período pós-parto, é importante que a investigação dos transtornos de ansiedade e TEPT também ocorra, dado que a comorbidade e multimorbidade são eventos frequentes, que compartilham os mesmos fatores de risco e afetam as mulheres em mais desvantagens sociais, como demonstrado nos resultados deste estudo. Adicionalmente, é importante que os profissionais de saúde estejam cientes da complexidade potencial da ocorrência da multimorbidade.

Embora este estudo tenha apresentado resultados consistentes com os de outras pesquisas, algumas limitações metodológicas podem ter influenciado os resultados. Uma possível limitação é inerente às pesquisas realizadas por telefone, estando sujeitas ao não atendimento das chamadas, números de telefone incorretos ou inexistentes e recusa em fornecer informações por telefone, o que ocasionou taxa de resposta de 64%. De qualquer modo, foram utilizadas algumas estratégias estatísticas para contornar o problema da não resposta. Além disso, é preciso observar que estudos que coletam informações psicossociais estão mais sujeitos aos fenômenos de aceitabilidade social e estigma por abordar temas sensíveis. Com isso, as participantes tendem a ser menos sinceras em suas respostas na tentativa de evitar o julgamento do entrevistador ou de tentar atender a uma expectativa social, podendo subestimar a prevalência.

Em contrapartida, destaca-se o fato de ser um estudo inovador, que pela primeira vez investigou a prevalência de TEPT associado ao parto e a coocorrência de sintomas de depressão e ansiedade em uma coorte de puérperas brasileiras. Adicionalmente, a coleta de informações por meio de várias fontes na linha de base (entrevista, cartão de pré-natal, prontuário materno e do recém-nascido e resultados de exames de ultrassonografia) abordou fatores de risco em vários domínios, permitindo testar diversas associações com os transtornos mentais investigados no *follow-up*.

## CONCLUSÃO

Este estudo destaca que o período do pós-parto é crítico para o diagnóstico de transtornos mentais e que pode cursar com quadros complexos em que sintomas de depressão, ansiedade e estresse se sobrepõem. É importante que os profissionais de saúde estejam cientes da ocorrência e coocorrência desses transtornos, bem como das possíveis consequências para a saúde da mulher e do recém-nascido, levando em conta que as desigualdades socioeconômicas e as barreiras do sistema de saúde podem exacerbar o ciclo de vulnerabilidade e saúde mental. O reconhecimento e a intervenção nesses fatores são fundamentais para a prevenção e o tratamento eficaz dos transtornos mentais perinatais.

## Data Availability

Os conjuntos de dados gerados e/ou analisados durante o estudo estão disponíveis com o autor correspondente mediante solicitação.
